# Within field spatial variation in methane emissions from lowland rice in Myanmar

**DOI:** 10.1186/s40064-015-0901-2

**Published:** 2015-03-26

**Authors:** Aung Zaw Oo, Khin Thuzar Win, Sonoko Dorothea Bellingrath-Kimura

**Affiliations:** Tokyo University of Agriculture and Technology, Graduate School of Agriculture, Department of International Environmental and Agricultural Science, Saiwaicho 3-5-8, Fuchu, Tokyo 183-8509 Japan

**Keywords:** Fertilizer, Lowland rice, Methane emission, Soil properties, Spatial variation

## Abstract

An assessment of within field spatial variations in grain yield and methane (CH_4_) emission was conducted in lowland rice fields of Myanmar. Two successive rice fields (1^st^ field and 2^nd^ field) were divided into fertilized and non-fertilized parts and CH_4_ measurements were conducted at the inlet, middle and outlet positions of each field. The results showed that CH_4_ emissions at non-fertilized parts were higher than those at fertilized part in both rice fields. The average CH_4_ emissions ranged from 8.7 to 26.6 mg m^-2^ h^-1^ in all positions in both rice fields. The spatial variation in CH_4_ emission among the positions was high in both rice fields with the highest emissions in the outlet of the 1^st^ field and the inlet of the 2^nd^ field. The CH_4_ emissions at these two positions showed 2 - 2.5 times higher than those at other positions in both rice fields. Stepwise regression analysis indicates that soil total carbon content is the primary factor for CH_4_ emission. The average CH_4_ emissions during rice growing season were 13.5 mg m^-2^ h^-1^ for the 1^st^ field and 15.7 mg m^-2^ h^-1^ for the 2^nd^ field. Spearman rank order correlation analysis showed that CH_4_ emission was significantly and positively correlated with soil temperature, surface water depth and negatively correlated with soil redox potential. The result indicated that high within field spatial variation in CH_4_ emissions required different site specific management practices to mitigate CH_4_ emissions in lowland paddy rice soil.

## 1. Introduction

Rice is the most important crop in Myanmar. In terms of rice growing area and production, Myanmar ranks seventh in the world (FAO 2010). The total area of rice cultivation is 8.06 million ha, among which 68% represents lowland rice cultivation areas (FAO 2010). Most of the major lowland rice growing areas such as the Ayeyarwady, Yangon and Bago Divisions are naturally provided with fertile deltaic alluvial soil and abundant monsoon rainfall. Irrigated lowland rice is one of the major rice ecosystems in these regions, especially in semi-rainfed areas. Rice fields in Myanmar are connected as successive fields in lowland areas with a few centimeters of difference in elevation. Even though the importance of paddy rice in Myanmar, basic information of the paddy rice cultivation such as spatial variability of soil properties and yield and its related methane (CH_4_) emission are still missing.

Spatial variation of CH_4_ emission from rice fields is regulated by a variety of agronomic and environmental factors, as well as the complex interactions of the whole system involving the rice plants, soil and atmosphere (Jean and Pierre 2001; Wang and Li 2002). Studies have shown variations in CH_4_ emission from continuously flooded rice soils in different locations with varying soil properties and climates (Kimura et al. 1991; Yang and Chang 2001; Kumar and Viyol 2009). Soil organic carbon (SOC) acts as a substrate for methanogens (Penning and Conrad 2007), thus it has significant correlation with CH_4_ production (Wassmann et al. 1998). In toposequence rice fields, the observed high rates of CH_4_ emission from middle and particularly bottom field positions were associated with their higher TN, TC and clay contents compared to the top field positions (Oo et al. 2013). Mitra et al. (2002) also observed that higher TN and TC stimulated CH_4_ production from rice soil. Xiong et al. (2007) reported that clay soil produced much more CH_4_ than loess soil during the flooding period. Soil properties vary highly even within a single field (Inman et al. 2005). Analysis of the spatial variability of CH_4_ emission with field is necessary to create inventory data for Myanmar.

It is well known that CH_4_ emission from paddy rice fields is a net product of CH_4_ production and oxidation. Methane emission from paddy rice fields during the growing season are significantly affected by water management, organic matter application, soil organic matter, C content, soil pH, preseason water status and climate (Yan et al. 2005). Beside the soil environmental factors, CH_4_ emissions from rice fields are also directly or indirectly affected by application of N and other nutrients (Schimel 2000). For example, at the plant or ecosystem level, ammonium-based fertilizers can stimulate rice plant growth, which may increase CH_4_ emission by providing more methanogenic substrates and enhancing the efficiency of CH_4_ transport to the atmosphere (Schimel 2000; Bodelier et al. 2000b; Zheng et al. 2006). Several field-scale studies have demonstrated that addition of N fertilizers increases CH_4_ emissions in rice soils (Banik et al. 1996; Shang et al. 2011). In contrast, others have observed that CH_4_ emissions were inhibited with N fertilizer (Xie et al. 2010; Dong et al. 2011). Application of phosphate (P) fertilizer may stimulate CH_4_ uptake in the soil (Zang et al. 2011) and inhibits the acetoclastic methanogenic activity in the rice rhizosphere (Conrad et al. 2000) which leads to inhibition of CH_4_ emission from rice soil. Application of potassium (K) fertilizer alleviates the soil reducing condition (Chen et al. 1997) and inhibits the CH_4_ emission from rice field (Babu et al. 2006). However, there are also studies that report no effect on CH_4_ emission due to K fertilizer (Wassmann et al. 1993). The application of P and K fertilizer inhibits CH_4_ emission from soil may be due to their effect on plant ventilation and root exudates (Conrad and Klose 2005). In Myanmar, common practice of fertilizer application for paddy rice is urea and ammonium sulfate as a source of nitrogen, triple super phosphate as a source of phosphorus and muriate of potash as a source of potassium. The effect of N, P and K fertilizers on CH_4_ emission from rice soil in Myanmar is uncertain.

Due to the interactive effects of soil, climatic and cultural factors, the uncertainty in estimating CH_4_ emission from rice fields is high. The upscaling of emission rates is hampered by this uncertainty and the pronounced spatial and temporal variations (Sass et al. 2002). There is an urgent need to evaluate the interaction between CH_4_ emission and rice production in a changing climate in order to estimate source strength (Neue et al. 1997) and provide a basis for future decisions regarding mitigation options. Extensive field measurement of CH_4_ emission is necessary to develop a reliable regional and global CH_4_ budget and identify effective mitigation measures, especially in area where no study on CH_4_ emission is conducted yet, such as in Myanmar. In this experiment, one year filed experiment was conducted to understand within field spatial variation in CH_4_ emission among the positions in the field related to water flow pattern and its related soil and soil environmental factors. The objective of this study was to assess the spatial variations in soil properties, plant performance and CH_4_ emissions from different positions within a field in relation to water flow pattern and mineral fertilizers in lowland rice of Myanmar.

## 2. Materials and methods

### 2.1. Study site and experimental design

**Figure 1:**
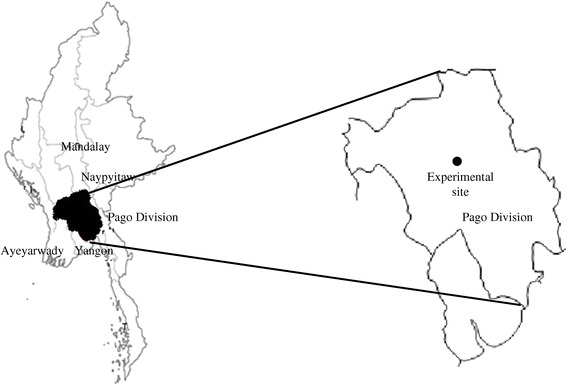
**Location of the experimental site in Kanyutkwin, Phyu City, Myanmar.**

**Figure 2:**
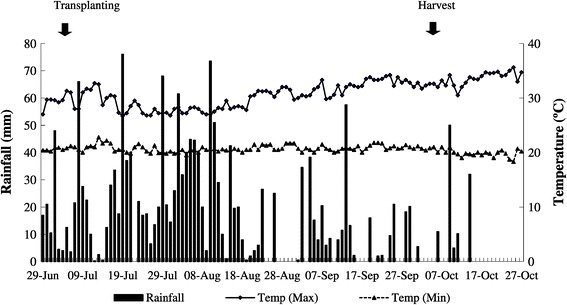
**Daily rainfall distribution, maximum and minimum temperatures during monsoon rice growing season for 2012 at experimental site of Kanyutkwin, Phyu City, Myanmar.**

The field experiment was carried out from June to November, 2012 during the monsoon rice growing season in Dawmakwin Village, Kanyutkwin, Pago Division, (18°48′43″ N, 96°43′57″ E), Myanmar (Figure 1). The field had been under rice (*Oryza sativa* L.) and black gram (*Vigna mungo*) rotation for 25 years. The soil was classified as a fluvisol (alluvial soil) (FAO/UNESCO, Food and Agriculture Organization of the United Nations/United Nations Educational and Cultural Organization 1974), which contained large amount of silt (Table 1). The weather in the study area is tropical monsoon climate with an annual rainfall of 2545 mm, and minimum and maximum temperatures of 19.6°C and 32.2°C, respectively, in 2012 (Figure 2).

**Table 1:** **Physico-chemical properties of the experimental soils at different positions within the field before transplanting of lowland paddy rice, 2012**

		**Sand**	**Silt**	**Clay**	**TN**	**TC**	**Organic matter (%)**	**pH**	**EC**
		**(%)**	**(%)**	**(%)**	**(g kg** ^**−1**^ **)**	**(g kg** ^**−1**^ **)**			**(ms m** ^**−1**^ **)**
**1** ^**st**^ **field**	Inlet	37.8 a	28.0 b	34.2 c	0.28 a	2.2 ab	5.1 a	6.0 bc	0.44 ab
	Middle	19.9 b	37.4 b	40.7 bc	0.32 a	2.0 b	5.3 a	6.4 a	0.41 b
	Outlet	10.8 c	44.5 ab	44.7 ab	0.33 a	2.7 a	5.5 a	6.2 ab	0.58 a
**2** ^**nd**^ **field**	Inlet	10.2 c	36.0 b	53.8 a	0.12 b	2.5 a	5.4 a	5.7 d	0.56 a
	Middle	8.3 c	48.7 a	39.0 bc	0.12 b	2.0 b	4.7 ab	5.7 d	0.39 b
	Outlet	9.7 c	52.0 a	38.4 bc	0.08 b	1.1 c	4.2 b	5.9 cd	0.32 b

Means with the same letter are not significant difference at 5% level by Fischer.

**Figure 3:**
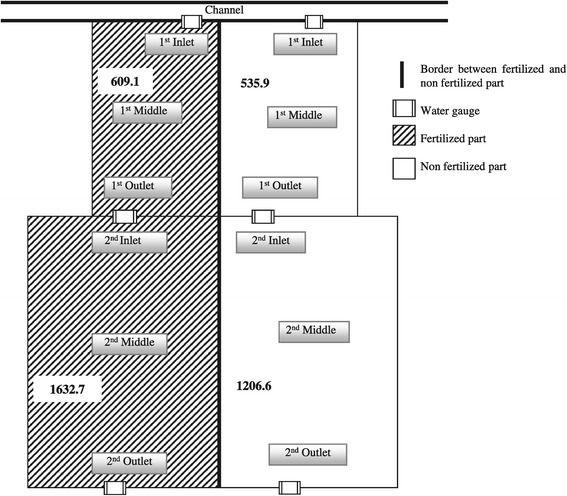
**Schematic representation of experimental layout with two successive lowland rice fields, Kanyutkwin, Myanmar (Area – m** ^**2**^ **).**

Two successive rice fields (hereafter referred to as the 1^st^ field and 2^nd^ field) covering a total of 0.5 ha were selected for this experiment (Figure 3). The 1^st^ field received water directly from the channel with a single inlet and water drained via a single outlet to the 2^nd^ field. The 2^nd^ field received water from a single inlet from the above-lying 1^st^ field and water drained via a single outlet to a lower-situated field. According to this water flow pattern, the field was divided into three positions: inlet, middle and outlet position.

The experiment was laid out in a split-plot design with three replications for each field (Figure 3). All fields were divided into two parts to produce two strips to separate fertilized and non-fertilized parts. Two sets of factors included in this experiment were as follows: with (+F) and without (-F) fertilizer application as the main plot, and different positions as a subplot. The positions were the inlet, middle and outlet for the two fields, referred to as the 1^st^ inlet, 1^st^ middle, 1^st^ outlet for the 1^st^ field, and the 2^nd^ inlet, 2^nd^ middle and 2^nd^ outlet for the 2^nd^ field. The applied chemical fertilizers were Nitrogen (N) 50 kg ha^-1^ (Ammonium sulfate), Phosphorus (P) 30 kg ha^-1^ (Triple superphosphate) and Potassium (K) 20 kg ha^-1^ (Muriate of potash) with two split applications according to the local recommendations by extension service. The first dressing was conducted at transplanting using 50% N, 100% P and 100% K of the total amount of fertilized applied. The second dressing contained the remaining 50% N which was applied at heading stage (60 days after transplanting).

The indica rice variety (O*ryza sativa* L. var. Sinthukha) was used in this experiment. Thirty-day-old seedlings were manually transplanted into the well-puddled fields. Rice seedlings were transplanted on June 29 and harvested on October 11, 2012. All management practices followed farmer practices. The fields were flooded 22 days before transplanting on June 7, 2011. The basal fertilizer was applied one day before transplanting. After transplanting, the fields were continuously flooded until 14 days before harvest because mid-season drainage was not successful due to continuous rainfall during that period.

### 2.2. Sample collection, soil parameters, and CH_4_ analysis

Methane fluxes were measured in triplicate at 10-day intervals from 7 days after transplanting (DAT) until harvest throughout the rice growing seasons, using the closed chamber method (Lu et al. 1999) at each point. The air inside the chamber was mixed by a fan at the top of the chamber. Gas samples were drawn from the chambers through a three-way stopcock using an airtight 50-ml syringe at 0, 15 and 30 minutes after closure. The air inside the chamber was thoroughly mixed by flushing the syringe three times before collecting the gas samples. The gas samples were then transferred to 10-ml vacuum glass vials with rubber stoppers and kept cool and dark until analysis. The temperature inside the chamber was recorded at the time of sampling using a micro-temperature thermometer (PC-9125, AS ONE Co., Tokyo, Japan). Methane concentrations in the collected gas samples were analyzed using a gas chromatograph equipped with a flame ionization detector (GC-8A, Shimadzu Corporation, Kyoto, Japan). The detector and column were operated at 180° and 80°C, respectively. Methane fluxes were calculated from the slope of a CH_4_ concentration vs. time regression when their linear correlation coefficients were significant at the 0.05 level.

Top-soil samples at a depth of 0–10 cm were taken before transplanting to analyze the physical and chemical properties of the soil. Soil particle analysis was performed using the pipette method (Gee and Bauder 1986), and soil organic matter contents were analyzed by the hydrogen peroxide method (Schultz et al. 1999). Total nitrogen (TN) and total carbon (TC) contents were analyzed using an NC analyzer (Sumigraph NC-80; Sumika Chemical Analysis Service Co., Japan). The soil pH was measured in the supernatant suspension of a 1:2.5 soil: water mixture using a portable pH meter equipped with a combined electrode (glass:Ag/AgCl, Horiba, Japan). The electrical conductivity of the soil water was measured in the supernatant suspension of a 1:5 soil : water mixture using an EC meter (OM-51, Horiba, Japan).

Soil temperature at a depth of 10 cm was recorded at the time of gas sampling. Water depth was also recorded among the positions at 10-day intervals throughout the growing seasons. The pH of the surface water was measured using a portable pH meter (D-51T, Horiba, Japan) equipped with combined electrode (glass:Ag/AgCl). The redox potential was recorded using a battery-operated Eh meter (D-51T, Horiba, Japan) by inserting the platinum electrode into the soil under investigation to a root-zone depth of 5 cm throughout the growing season. Mean value of redox potential was shown in the result using the raw millivolt data, not used any correction factor. Plant height and tiller number were recorded as growth parameters, and grain yield was determined from a 1-m^2^ sampling area at harvest and was expressed as unhulled rice at 14% moisture content. Aboveground straw weight was determined after drying the plant materials at 80°C for two days.

### 2.3. Statistical analysis of data

Statistical analysis was performed using the CropStat 7.2 statistical software program. The treatment mean comparison was tested at the 5% level of probability using the least significant difference (LSD) test by Fischer. Comparison of rate and cumulative CH_4_ flux was performed separately for fertilizer and position effect. Stepwise multiple regression analysis was performed to determine relationship between soil properties and CH_4_ emission. Spearman rank order correlation analysis was done using the SigmaPlot 11.0 statistical software program.

## 3. Results

### 3.1. Soil environmental factors

Sand was the dominant type with 37% in the 1^st^ inlet position (Table 1). Silt content showed an increasing trend from the 1^st^ inlet to 2^nd^ outlet positions. The highest clay content was observed in the 1^st^ outlet and 2^nd^ inlet followed by the 2^nd^ middle and 1^st^ middle positions. Soil TC content differed significantly (p < 0.01) among positions and ranged from 1.1 to 2.5 g kg^-1^ soil in both experimental fields. The 1^st^ outlet and 2^nd^ inlet showed significantly (*p <* 0.05) higher TC content than that of other positions. Soil TN content and soil pH were higher in all positions (1^st^ inlet, 1^st^ middle and 1^st^ outlet) of the 1^st^ field compared to those of the 2^nd^ field. High organic matter and EC were observed in all positions of the 1^st^ field and 2^nd^ outlet of the 2^nd^ field.

**Figure 4:**
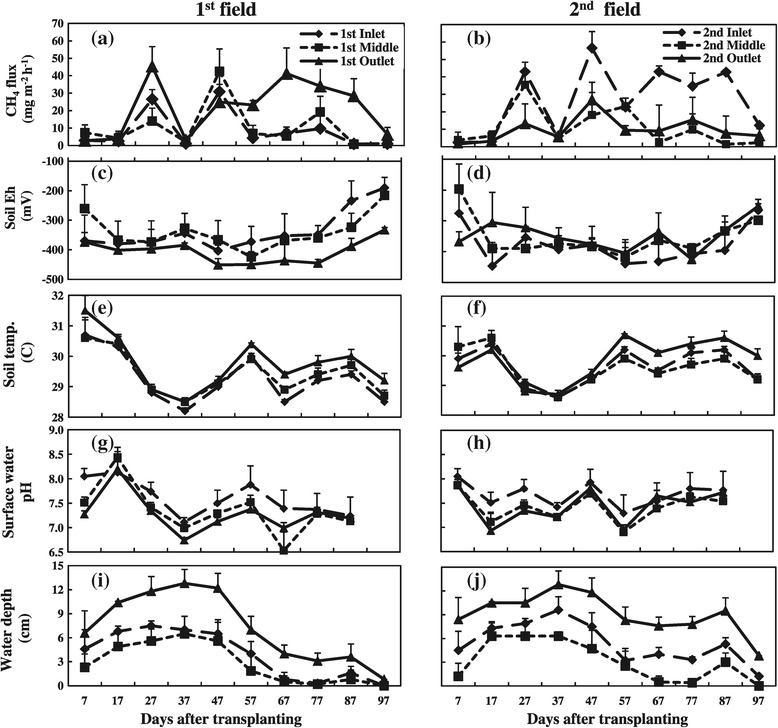
**Seasonal changes in (a) and (b) CH**_**4**_ **flux, (c) and (d) soil Eh, (e) and (f) soil temperature, (g) and (h) surface water pH and (i) and (j) surface water depth among the positions of 1** ^**st**^ **and 2** ^**nd**^ **field, respectively, during rice growing season (Bars-standard deviation).**

Soil redox potential (Eh) was as low as -180 mV at one week after transplanting and remained at a low level throughout the growing season (Figure 4c and d). Soil Eh of the 1^st^ outlet and 2^nd^ inlet positions tended to decrease faster and was lower than that of other positions, especially from 47 DAT to the end of the growing period. Soil temperature ranged from 28 to 32°C (Figure 4e and f). It was higher at the beginning and then decreased to the lowest value at 37 DAT due to continuous rain and cloudy conditions. The soil temperature then increased gradually and remained less variable until the end of the growing period. Among the positions, the 1^st^ outlet, 2^nd^ inlet and 2^nd^ outlet positions exhibited a higher soil temperature than that of other positions, especially during the middle and late growing periods. Surface water pH ranged between 6.5 and 8.5 throughout the growing season (Figure 4g and h). Significant (*p <* 0.05) differences in surface water depth among positions were observed throughout the growing season (Figure 4i and j). The 1^st^ and 2^nd^ outlets exhibited the highest water depths, followed by the 1^st^ and 2^nd^ inlets, and the lowest depth was found for both middle positions.

### 3.2. Influence of position on seasonal variation in CH_4_ emission

Methane emission from all positions in both fields generally showed two peaks during the rice growing season (Figure 4a and b). The first peak was found at 27 DAT (tillering stage), which was followed by a sudden drop at 37 DAT. The second peak occurred at 47 DAT (maximum tillering stage) for the 1^st^ inlet, 1^st^ middle, 2^nd^ middle and 2^nd^ outlet positions, which then decreased towards the end of the growing period. The 1^st^ outlet also showed an emission peak at 47 DAT and maintained a high emission to the end of the growing period. The 2^nd^ inlet showed a maximum peak at 67 and 87 DAT (booting and flowering stages), and exhibited a high emission in the late growing period.

Methane emission showed significant (*p <* 0.01) differences among positions in both rice fields (Table 2). The average rate and cumulative CH_4_ emission during the rice growing season exhibited the following order of magnitude: 2^nd^ inlet > 1^st^ outlet > 2^nd^ middle > 1^st^ middle > 2^nd^ outlet > 1^st^ inlet. The highest average CH_4_ emission was 21.3 and 26.6 mg CH_4_ m^-2^ h^-1^ in the 1^st^ outlet of the 1^st^ field and 2^nd^ inlet of the 2^nd^ field, respectively, and the lowest value of 8.7 mg CH_4_ m^-2^ h^-1^ was recorded for the 1^st^ inlet position of the 1^st^ field. The 1^st^ outlet and 2^nd^ inlet were 2 to 2.5 times significantly higher than that of other positions in both rice fields. The average CH_4_ emission rate of all positions in the 1^st^ field was 13.5 mg CH_4_ m^-2^ h^-1^, which did not differ statistically from that of the 2^nd^ field (15.7 mg CH_4_ m^-2^ h^-1^).

**Table 2:** **Influence of fertilizer and positions on rate and cumulative CH**_**4**_ **flux, plant growth and yield of lowland rice**

			**CH**_**4**_ **flux rate (mg m** ^**−2**^ **h** ^**−1**^ **)**	**CH**_**4**_ **flux Cumulative (g m** ^**−2**^ **)**	**Tiller number**	**Plant height (cm)**	**Grain (g m** ^**−2**^ **)**	**Straw (g m** ^**−2**^ **)**
**1** ^**st**^ **field**	**Treatment**	-F	15.5 a	38.7 a	9.0 b	106.9 a	501.9 a	608.8 b
		+F	11.4 a	28.5 a	9.9 a	111.2 a	528.2 b	631.1 a
	**Position**	Inlet	8.7 b	21.7 b	9.0 a	111.5 a	532.7 a	636.0 a
		Middle	10.4 b	26.0 b	9.7 a	106.5 a	507.8 b	598.5 b
		Outlet	21.3 a	53.2 a	9.5 a	109.0 a	534.7 a	630.0 a
**2** ^**nd**^ **field**	**Treatment**	-F	16.8 b	41.9 b	8.0 b	101.5 b	424.9 b	544.1 b
		+F	11.8 a	29.5 a	8.9 a	113.6 a	493.0 a	580.7 a
	**Position**	Inlet	26.6 a	66.4 a	8.9 a	105.3 a	475.2 a	576.3 a
		Middle	10.8 b	27.0 b	7.8 ab	98.4 b	462.7 ab	532.3 b
		Outlet	9.8 b	24.5 b	7.7 b	104.8 a	449.0 b	534.8 b

-F and +F stand for non-fertilized and fertilized part, respectively.

Means with the same letter are not significant difference at 5% level by Fischer.

**Figure 5:**
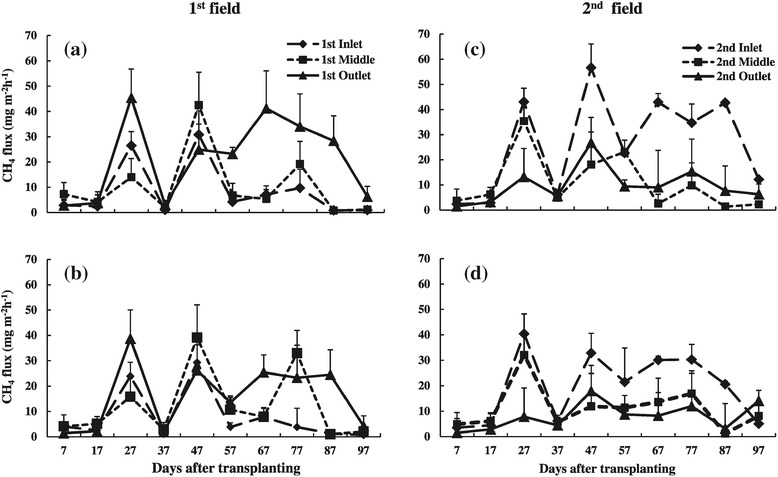
**Seasonal changes in CH**_**4**_ **fluxes in non-fertilized (a and c) and fertilized parts (b and d) among the positions of 1** ^**st**^ **and 2** ^**nd**^ **field during rice growing season, respectively (Bars-standard deviation).**

Seasonal variation in CH_4_ emission from fertilized and non-fertilized parts showed similar trends and patterns throughout the growing season (Figure 5). The average rate and cumulative CH_4_ emission from fertilized parts was significantly (*p <* 0.05) lower than that from non-fertilized parts in both rice fields (Table 2). Average CH_4_ emissions for non-fertilized and fertilized parts were 15.5 and 11.4 mg CH_4_ m^-2^ h^-1^ in the 1^st^ field and 16.8 and 11.8 mg CH_4_ m^-2^ h^-1^ in the 2^nd^ field, respectively.

### 3.3. Plant growth and crop yield

There were significant (*p <* 0.05) differences in tiller number and plant height among the positions only in the 2^nd^ field (Table 2). All positions in the 1^st^ field showed a higher tiller number and plant height compared to that of any position in the 2^nd^ field. Significant (*p <* 0.05) differences in straw and grain yield were observed among the positions in both rice field. Higher rice straw and grain yields were found for all positions in the 1^st^ field, while the lowest grain yield was observed in the 2^nd^ outlet position. Fertilized parts showed significant (*p <* 0.05) higher tiller number, straw and grain yield in both rice fields while plant height was observed only significant (*p <* 0.05) in the 2^nd^ field due to fertilization.

### 3.4. Influence of soil properties and soil environmental factors and plant growth on CH_4_ emission

To determine the controlling factors of CH_4_ emission, soil properties from Table 1 were used in the stepwise multiple regression. According to analysis result, five soil characteristics; organic matter content, soil EC, clay, sand and TC content were found to greatly affect (p < 0.05) and account for 97% of the variance in CH_4_ emission in the 1^st^ field. The equation for the stepwise multiple regression is: CH_4_ emission = 65.929 + (4.517 * OM (%)) + (41.686 * EC) - (0.401 * Clay (%)) - (0.476 * silt (%)) - (12.079 * pH) + (2.265 * TC (g kg^-1^)**)**. In the 2^nd^ field, only two soil characteristics; clay and TC content were found to greatly affect (p < 0.001) and account for 76% of the temporal variability in the CH_4_ emission. The equation for the stepwise multiple regression is: CH_4_ emission = -11.381 + (0.425 * clay (%)) + (6.196 * TC (g kg^-1^)).

Surface water pH was significantly correlated with CH_4_ emission for both rice fields (Table 3). Surface water depth showed a significant and positive correlation with CH_4_ emission in the 1^st^ field, but there was no significant correlation for the 2^nd^ field. Significant negative correlations between CH_4_ emission rate and soil Eh were observed in both rice fields. Soil temperature was significantly and positively related with CH_4_ emission only in the 1^st^ field. The CH_4_ emission rate was positively correlated with TC content in both rice fields. No correlation was observed between CH_4_ emission and TN, or plant growth and yield parameters, for either of the rice fields.

**Table 3:** **Spearman rank order correlation between CH**_**4**_ **emission rate and soil and plant parameters of lowland rice**

	**Soil temp.**	**Surface water pH**	**Water depth**	**Soil Eh**	**TN**	**TC**	**Grain**	**Straw**
1^st^ field	0.51*	0.74*	0.54*	−0.67**	0.34^ns^	0.55*	0.04^ns^	0.03^ns^
2^nd^ field	0.29^ns^	0.89**	0.04^ns^	−0.56**	0.45^ns^	0.57*	0.12^ns^	0.24^ns^

**, * and ns stand for significant at 1%, 5% and non significant, respectively.

## 4. Discussion

### 4.1. Average CH_4_ emission

Methane emissions showed two peaks in both rice fields, while the trend of the position differed between the fileds (Figure 4a and b). The first emission peak was observed at 27 DAT (active tillering stage), which might be associated with microbial decomposition of native organic matter under high temperature (Holzapfel et al. 1986). Higher CH_4_ emission at the tillering stage was generally due to lower rhizospheric CH_4_ oxidation and more effective transport mediated by rice plants (Suryavanshi et al. 2012). Li et al. (2011) also suggested that this was caused by fermentation of easily degradable soil organic matter and flood conditions for methanogenesis in the soil after transplanting.

The sudden drop of CH_4_ emission at 37 DAT was probably due to low soil temperature and continuous rainfall during this period (Figure 2). The temperature did not drop as low as to inhibit methanogen activity, but it affected the diffusion of CH_4_ from the water to the air concentrations according to Henry`s law (Carroll 1991). More gas can be dissolved as entrapped bubbles at low temperature and leads to decrease in the apparent emission. The rain also did not directly influence the methanogen activity, but due to the associated change in the air pressure. Gaseous CH_4_ pool in the water can be released as bubbles if air pressure drops (Tokida et al. 2012). The static closed chamber techniques often failed to catch sudden changes in emissions and thus, all points showed low emission at 37 DAT.

The second emission peak occurred at maximum tillering (47 DAT) and the late growing period. This could be attributed to high soil temperature and decomposition of soil organic matter and decaying plant residues from shed leaves and root turnover. Methane emissions during late growing periods might have been associated with the higher availability of root exudates or decaying plant residues for methanogenic bacteria in the rice rhizosphere, and the highly reduced conditions in this rhizosphere (Mitra et al. 2005).

The seasonal average CH_4_ emission rate ranged from 8.7 to 26.6 mg CH_4_ m^-2^ h^-1^ over the rice growing season (Table 2). This is higher than the IPCC (1996) default value of CH_4_ emission; 8.3 mg CH_4_ m^-2^ h^-1^ from irrigated rice fields for Myanmar. Lowland-upland rotation usually results in low CH_4_ emissions; such as 1.4 mg CH_4_ m^-2^ h^-1^ averaging over the rice growing season in rice-wheat rotation (Zou et al. 2004) or an average CH_4_ emission during rice growing season of 3.4 mg CH_4_ m^-2^ h^-1^ after upland crops such as mustard, chickpea or blackgram (Adhya et al. 2000). The range of cumulative seasonal CH_4_ emissions in this study was 21.2 to 66.4 g CH_4_ m^-2^ (Table 2). This is comparatively higher than that reported for toposequence rice fields cultivating double-cropping paddy rice in Northwest Vietnam, which ranged from 7.4 g m^-2^ to 37.2 g CH_4_ m^-2^ (Oo et al. 2013). The rather higher emissions might be due to the difference in elevation between the fields, while in our study a difference of only a few centimeters existed between the fields with poor drainage, continuous flooding and high soil temperature throughout the growing season. Differences in CH_4_ emissions between two locations were also shown by Zhang et al. (2009), who reported high spatial variability in CH_4_ emissions from rice fields in the Taihu Lake region of China, and demonstrated higher annual CH_4_ emissions on the plains compared to the hilly regions.

The fields in our study were located in a lowland area with poor field drainage conditions. The installation of drainage pipes or other techniques to improve drainage are seldom found in Myanmar and almost all paddy fields in Myanmar are under similar situation with the current field condition. In addition, the fields were continuously flooded throughout the rice growing season. Mid-season drainage could not be conducted due to frequent rainfall (Figure 2). The soil Eh was kept low (Figure 4a and b), and the high temperature favored high CH_4_ emission. Since a wide area of paddy fields in Myanmar are under similar condition as our field, the current CH_4_ emission from Myanmar may be highly underestimated.

### 4.2 Influence of mineral fertilizer on CH_4_ emission

Many paddy rice fields in Myanmar rely on mineral fertilizers to increase crop yields (FAO 2010). Nitrogen fertilizers stimulate crop growth and provide more C substrates via organic root exudates and sloughed-off cells to methanogens for CH_4_ production (van der Gon et al. 2002; Inubushi et al. 2003). Addition of N fertilizer increased CH_4_ emissions in rice soil due to the stimulation of methanogens by greater production of crop biomass under N fertilization (Banik et al. 1996) and Shang et al. 2011). In contrast, N fertilizer has also stimulated the activities of methanotrophs that resulted in greater CH_4_ oxidation (Bodelier et al. 2000a, b). Methane emission rate from ammonium sulfate treatment was the lowest, followed by ammonium chloride treatment, and then urea treatment (Kimura 1992). Another study reported that CH_4_ emission was 30–50% lower following application of ammonium sulfate compared to urea-treated rice plots (Cai et al. 1997). For P and K fertilizers, reduction of CH_4_ was found in several studies (Rao et al. 1986, Adhya et al. 1998, Babu et al. 2006), while also no effect was also found (Wassmann et al. 1993). In this study, the results showed that application of ammonium sulfate as N source, triple superphosphate as P source and potassium sulfate as K source inhibited the rate and cumulative CH_4_ emissions from rice soils by 26.5 and 29.8% in the 1^st^ field and 2^nd^ field respectively when compared with non-fertilized treatment (Figure 5 and Table 2). A similar result was reported by Datta et al. (2013) and Yang et al. (2010). Sulfate-containing fertilizers are known to decrease CH_4_ emission as a result of competition between sulfate-reducing bacteria and methanogens for hydrogen and acetate substrates (Hori et al. 1990, Schütz et al. 1989). Significant reduction in CH_4_ emission due to fertilization could be either due to ammonium sulfate, or due to P and K fertilizers in this study.

### 4.3. Influence of different positions and soil environmental factors on CH_4_ emission

Many studies have reported that soil properties of paddy rice have a strong influence on CH_4_ emission from rice fields (Mitra et al. 2005; Oo et al. 2013). This experiment showed that the soil variability affects significantly the CH_4_ emissions not only among different fields, but also within a field (Figure 4a and b). Spatial variability of CH_4_ emissions among toposequence positions was related to transportation and deposition of organic-rich sediment materials within toposequence rice fields in Viet Nam (Oo et al. 2013). In the current study, the observed high rates of CH_4_ emission from the 1^st^ outlet and 2^nd^ inlet positions were associated with a high clay, TC and organic matter content (Tables 1 and 3). Schmitter et al. (2010) reported that the increase of SOC was related to an increase of clay and silt fractions which point to transportation of organic rich sediment material by the irrigation channel. In the current study, it showed clearly that most of the sediments deposited in the 1^st^ field (Table 1). As a result, sand showed decreasing trend with the highest content in 1^st^ inlet, silt showed increasing trend with the highest in 2^nd^ outlet and the highest clay was observed in 1^st^ outlet and 2^nd^ inlet positions. Since irrigation water velocity was low, the condition between 1^st^ outlet and 2^nd^ inlet was similar. Both positions showed high clay, TC, soil EC, organic matter content and low sand content (Table 1).

Higher clay and TC contents favor methanogenic activities (Mitra et al. 2002; Xiong et al. 2007; Gaihre et al. 2011) as also confirmed by the positively correlation of TC content and CH_4_ emission in this study (Table 3). Higher TC content stimulated CH_4_ production in rice soil (Mitra et al. 2002, Oo et al. 2013). Another reason for the high rates of CH_4_ emission from the 1^st^ outlet and 2^nd^ inlet might be the lower Eh values due to a high water depth in these positions (Figure 4i and j), which were negatively correlated with CH_4_ emission in this study (Table 3). Soil with a high clay and TC content exhibits negative Eh values within two weeks after submergence, and thereafter becomes more negative than soil with a lower C content (Xiong et al. 2007). A more rapid decrease in Eh after flooding and subsequent stability due to the high C content in the outlet of the 1^st^ field and inlet of the 2^nd^ field might explain the greater CH_4_ production. Stepwise multiple regression analysis was used to identify key factors regulating CH_4_ emission from soils and the results showed that a series of soil factors could affect CH_4_ emission from both rice fields. However, there was only one common factor; TC content regulating CH_4_ emission from both rice soils. The positive relationship of CH_4_ emission with TC content has been widely reported (Mitra et al. 2002, Xiong et al. 2007, Gaihre et al. 2011, Oo et al. 2013). Although clay content appears in both regression equations for the two fields, clay content is not the primary factor for controlling CH_4_ emission due to the opposite values in equations in this study.

In this study, seasonal variation in CH_4_ emission was influenced by soil environmental factors such as soil Eh, surface water pH, soil temperature and surface water depth (Figure 4c-j). The soil redox potential declined after flooding and fluctuated between – 150 and – 450 mV in both fields throughout the growing season (Figure 4c and d). Negative correlations between CH_4_ emissions and soil Eh existed in both rice fields (Table 3). Yagi and Minami 1990 reported that the critical values of soil Eh for initiation of Eh from -100 to -200 mV. The Eh range in this study was considerable more negative than the critical value and the low Eh easily led to higher methane production. Although positive correlation between soil temperature and CH_4_ emission was observed only in 1^st^ field (Table 3), the temperature range in this experiment fall within the optimum temperature for methanogens ranged from 25 to 37°C (Schütz et al. 1990). There were significant correlation between CH_4_ emissions and surface water pH in both 1^st^ and 2^nd^ fields. High standing water pH might favor CH_4_ emission in this study. Significant correlation between CH_4_ emission and surface water depth was observed only in the 1^st^ field but not the case in the 2^nd^ field (Table 3). Under flooded condition, negative redox status was already established (Figure 4c and d) and variation in surface water depth during growing season might not have affected on big differences of soil redox status (Figure 4i and j). Gaihre et al. 2011 reported that the contribution of floodwater depth was not significant in their study because they maintained the field continuously flooded, the small variation in depth might not have affected CH_4_ emissions to a large extent. High spatial variation in CH_4_ emission within the rice field in this experiment was mainly due to variation in TC content and soil environmental factors among the positions. Our analysis was focused on the effect of farmer management practice on within field spatial variation in CH_4_ emission and we highlighted that there were high variations in CH_4_ emissions among the positions and its influencing factors from paddy rice soil. Pandey et al. (2014) also showed one year field experiment to examine whether different organic amendments in combination with the safe alternate wetting and drying has the potential to suppress on greenhouse gas emissions from rice paddies. To develop effective mitigation strategies, further work is needed to investigate small and large scale spatial variations in CH_4_ emission from rice soil for different locations in lowland area of Myanmar.

## 5. Conclusion

High spatial variations in grain yield and CH_4_ emissions among the positions were found in two successive rice fields. The positions near the channel showed better soil fertility status as well as better growth performance in this study. The outlet of the 1^st^ field and inlet of the 2^nd^ field showed highest CH_4_ emissions. The data strongly indicated that high CH_4_ emissions were due to high TC content with low redox potential in these positions. Within field spatial variation in CH_4_ emission was related with soil TC content, and soil environmental factors which showed differences among the positions in both rice fields according to water flow pattern. Application of mineral fertilizers reduced CH_4_ emissions from paddy rice soil as compared to non-fertilized parts in both rice fields. Current mineral fertilizers management practice such ammonium based N fertilizer, triple superphosphate and muriate of potash is an effective way to reduce CH_4_ emission from lowland rice fields but site specific management practices should be adopted for different positions to increase grain yield and mitigate CH_4_ emission from lowland rice in Myanmar.
